# Inflammatory and Hemostatic Markers in COVID-19 Patients with Arterial Thrombosis Are Significantly Lower at Hospital Admission than in COVID-19 Patients without Thrombosis

**DOI:** 10.3390/v14112330

**Published:** 2022-10-24

**Authors:** Miguel de Oliveira, Francisco Cubal, Maria Coutinho, Mónica Pereira, Eugénia Cruz, Sara Morais

**Affiliations:** 1 Unidade de Trombose e Hemostase, Serviço de Hematologia Clínica, Hospital de Santo António (HSA), Centro Hospitalar Universitário do Porto (CHUP), 4099-001 Porto, Portugal; 2 Serviço de Hematologia Clínica, Centro Hospitalar de Trás-os-Montes e Alto Douro, 5000-508 Vila Real, Portugal; 3 Unidade Multidisciplinar de Investigação Biomédica, Instituto de Ciências Biomédicas Abel Salazar, Universidade do Porto (UMIB/ICBAS/UP), 4050-313 Porto, Portugal

**Keywords:** COVID-19, arterial thromboembolism, venous thromboembolism, inflammatory markers, hemostatic markers

## Abstract

Patients with Coronavirus disease 2019 (COVID-19) are at increased risk of venous thromboembolism (VTE); however, data on arterial thromboembolism (ATE) is still limited. We report a case series of thromboembolic events (TE) in 290 COVID-19 patients admitted between October and December 2020 to a Portuguese hospital. Admission levels of various laboratory parameters were evaluated and compared between COVID-19 patients with (TE) and without thrombotic events (non-TE). The overall incidence of isolated ATE was 5.52%, isolated VTE was 2.41% and multiple mixed events was 0.7%. A total of 68% events were detected upon admission to the hospital with 76% corresponding to ATE. Admissions to the Intensive Care Unit were higher in patients with TE, when comparing with the non-TE group (44% vs. 27.2%; *p* = 0.003). Patients with ATE presented significantly lower levels of CRP (*p* = 0.007), ferritin (*p* = 0.045), LDH (*p* = 0.037), fibrinogen (*p* = 0.010) and higher monocyte counts (*p* = 0.033) comparatively to the non-TE patients. These results point to an early occurrence of TE and an increased incidence of ATE over VTE. The less prominent inflammation markers in patients with TE and the early presence of TE in patients with otherwise no reason for hospitalization, may suggest a direct role of SARS-CoV-2 in the thrombotic process.

## 1. Introduction

The Coronavirus disease of 2019 (COVID-19) has been associated with endothelial injury, complement-induced blood clotting, systemic microangiopathy and a dysregulated inflammatory response manifesting as a cytokine storm, ultimately leading to a highly prothrombotic state [1]. However, it remains unclear whether the direct infection of the endothelium or the intense inflammatory response to the Severe Acute Respiratory Syndrome Coronavirus 2 (SARS-CoV-2) virus predominates in originating the thrombotic tendency observed [2].

Accumulating evidence points to an increased incidence of venous thromboembolism (VTE) [3], which has led to numerous efforts towards optimizing thromboprophylaxis in this setting [4,5]. The association between COVID-19 and arterial thrombotic events (ATE) is less well established. While there is evidence towards an increased incidence of cerebrovascular events [6], there seems to be a reduced incidence regarding cardiovascular events [7,8]. Environmental factors and changes in healthcare accessibility patterns are likely contributors [9]. Several laboratory parameters were also associated with disease severity and thrombosis development, pointing to the importance of the individual inflammatory and hemostatic response in the outcomes [10,11,12].

In the northern region of Portugal, in the last trimester of 2020, the incidence of COVID-19 peaked, with 1313 new cases per 100,000 population, which led to an enormous pressure on the healthcare system [13]. During this period, there was a perceived increased incidence of ATE, namely, ischemic strokes and ST-elevation myocardial infarctions (STEMI) and a lower incidence of VTE in our COVID-19 population, contrary to previous reports in the literature. 

In order to clarify the incidence of both arterial and venous thrombosis, we retrospectively evaluated clinical records of hospitalized COVID-19 patients and searched for thrombotic events (TE). Concurrently, we investigated the association of several inflammatory and coagulation laboratory parameters at hospital admission with the establishment of both venous and arterial events.

## 2. Materials and Methods

We performed a retrospective analysis of the clinical records of COVID-19 patients admitted in our tertiary hospital, between October and December 2020. Only patients with SARS-CoV-2 infection confirmed with Polymerase Chain Reaction testing (PCR) were considered. A total of 290 patients, consecutively selected from the laboratory coagulation parameters registries, were included. Clinical records were searched for TE, comorbidities and both prior and in-hospital antithrombotic therapies. Disease severity was defined according to the highest level of care (general ward vs. intensive care unit (ICU)). Patient outcomes, i.e., hospital discharge or death, were also recorded. Hospital admission levels of fibrinogen, D-dimers, C-reactive protein (CRP), ferritin, lactate dehydrogenase (LDH), Creatine Kinase (CK), myoglobin and complete blood counts were evaluated. A control group of COVID-19 patients without TE (non-TE group) was selected for comparisons of laboratory parameters with the TE group, matching age, sex, comorbid conditions and disease severity (see Table 1). 

### 2.1. Laboratory Determinations 

Peripheral blood samples were collected by venipuncture into tubes containing sodium citrate for coagulation tests, serum separator tubes with silica/gel for biochemical assays and tripotassium ethylene diamine tetracetic acid (EDTA-K3) for blood counts. Complete blood counts were obtained using the automated hematological analyzer Sysmex XE-2100 (TOA Medical Electronics, Kobe, Japan). All the coagulation tests (Prothrombin Time (PT), Activated Partial Thromboplastin Time (aPTT), Fibrinogen and D-dimer) were performed in an ACLTOP (Werfen, Orangeburg, NY, USA). Fibrinogen was measured according to the Clauss method and D-dimer was measured by latex-based assay (Werfen). Assays to determine the levels of CRP, ferritin, LDH, CK and myoglobin were performed on Cobas automated analyzer (Roche, Basel, Switzerland). The tests were performed according to each manufacturer’s instructions.

### 2.2. Statistical Analysis

Statistical analysis was conducted using IBM SPSS^®^, version 26. Continuous variables were expressed as mean ± standard deviation (SD) if they were normally distributed according to the Shapiro–Wilk test, or, if not, as median (interquartile range, IQR). Categorical variables were expressed as percentage (%) and frequency (n = #). Comparisons between TE and non-TE groups were conducted using the Kruskal–Wallis H test. A *p* value of <0.05 was considered statistically significant.

## 3. Results and Discussion

We conducted a retrospective analysis to identify the prevalence of both ATE and VTE in COVID-19 patients during the peak of the SARS-CoV-2 pandemic in Portugal. 

A total of 290 COVID-19 patients were included, with a gender distribution of 179 males to 111 females and a median age 70-years-old (IQR 58–78). Seventy-nine patients were admitted to the ICU and the remaining 211 to the general ward. Twenty-five patients developed TE and twenty-four patients without thrombosis were selected as control group (non-TE). There were no statistically significant differences between TE and non-TE patients, in terms of age, gender distribution and associated comorbidities (Table 1).

In this series of patients, 25 out of 290 patients (8.6%) developed TE (Table 2). Of these, 16/25 (64%) developed arterial thrombosis, corresponding to 11 ischemic strokes (44%) and 5 STEMI (20%); 7 out of 25 patients (28%) developed venous thrombosis, corresponding to 4/25 (16%) pulmonary embolisms and 3/25 (12%) other forms of VTE (1 cerebral venous sinus thrombosis, 1 deep leg vein thrombosis and 1 superior vena cava thrombosis). A small percentage of patients, 8% (n = 2), had multiple events, both arterial and venous. 

The overall incidence of isolated ATE was 5.52%, isolated VTE was 2.41% and multiple mixed events was 0.7%. Most of the events, 17/25 (68%) were detected upon admission to the hospital, and the majority of them, 13 events, corresponded to ATE. Among TE occurring in hospitalized patients, 87.5% occurred despite anticoagulant therapy. These patients were on standard prophylactic enoxaparin dosing regimen (40 mg, subcutaneous, once per day).

There is a much lower prevalence of VTE in our population when compared to the available literature, with reports of up to 17% [3]. There are two factors worth considering that likely contribute to this. First, only symptomatic VTE were included in this our analysis, as no routine screening of VTE/PE was performed. While a previous extended retrospective study that did not employ routine screening found a prevalence of 3.1% [14], a systematic review and meta-analysis estimated the overall VTE prevalence in patients without routine screening as 9.5%, although the authors suggested that the large proportion of ICU patients in the analyzed studies likely led to an overestimation of the prevalence [15]. Second, all hospitalized COVID-19 patients are started on either standard dose thromboprophylaxis with enoxaparin or intermediate dose in high-risk patients, at admission, unless an absolute contraindication is present. There is considerable heterogeneity in thromboprophylaxis strategies in the available literature, which contributes to the widely different reported VTE prevalence estimates. 

ATE presented with a twofold higher incidence of VTE in our analysis, and interestingly, most cases were upon hospital admission. When looking at the two different forms of ATE, we found a higher stroke incidence, 3.8%. A recent meta-analysis with a total of 108,571 patients found an incidence of 1.9%. The incidence of STEMI was 1.7%, which is also higher than the findings on other studies [8,9]. A total of 35.3% (n = 6) of the ATE patients were already on antiplatelet therapy prior to the event. A retrospective observational study found no evidence for an effect of antiplatelet therapy prior to COVID-19 infection on mortality or a protective effect against ATE [16].

Admissions to the ICU were higher in patients with TE, when comparing with the non-TE group (44% vs. 27.2%; *p* = 0.003). TE patients also presented with a higher mortality rate, although not significant. (28% vs. 20.76%; *p* = 0.402).

Inflammatory and hemostatic laboratory parameters evaluated at hospital admission, along with comparisons between different groups of patients, are presented in Table 3.

Inflammation/hemostasis parameters assessed at hospital admission were increased in COVID-19 patients but not to the same extent in the different groups (TE and non-TE). Interestingly, patients with non-TE presented significantly higher levels of fibrinogen and CRP when compared with the TE patients. 

Non-TE patients presented with significantly higher levels of fibrinogen and CRP when compared with the TE patients. D-dimers, as expected, were significantly higher in TE patients when compared with non-TE group. Further subgroup analysis revealed that D-dimers had the highest levels among VTE patients, constituting the only statistically significant parameter when compared with the non-TE group, which is in line with previous observations [15]. When comparing ATE and VTE, significantly lower LDH levels were observed on ATE. 

The most remarkable results arise when comparing those in the ATE group with the non-TE. Patients with arterial events, despite having elevated inflammatory/hemostasis markers, have significantly lower elevation comparatively to non-TE patients (significantly lower levels of CRP, ferritin, LDH and Fibrinogen). This less obvious inflammatory status of ATE patients does not support the proposed mechanism of a hyperimmune response and cytokine storm mediating endothelial and vascular disruption as a cause of arterial thrombosis [1]. It suggests that the SARS-CoV-2 virus may have a direct cytopathic role in the endothelium, early in the disease course, at least in patients with ATE. This is supported by findings that show viral tropism towards endothelial cells [17]. 

**Figure 1:**
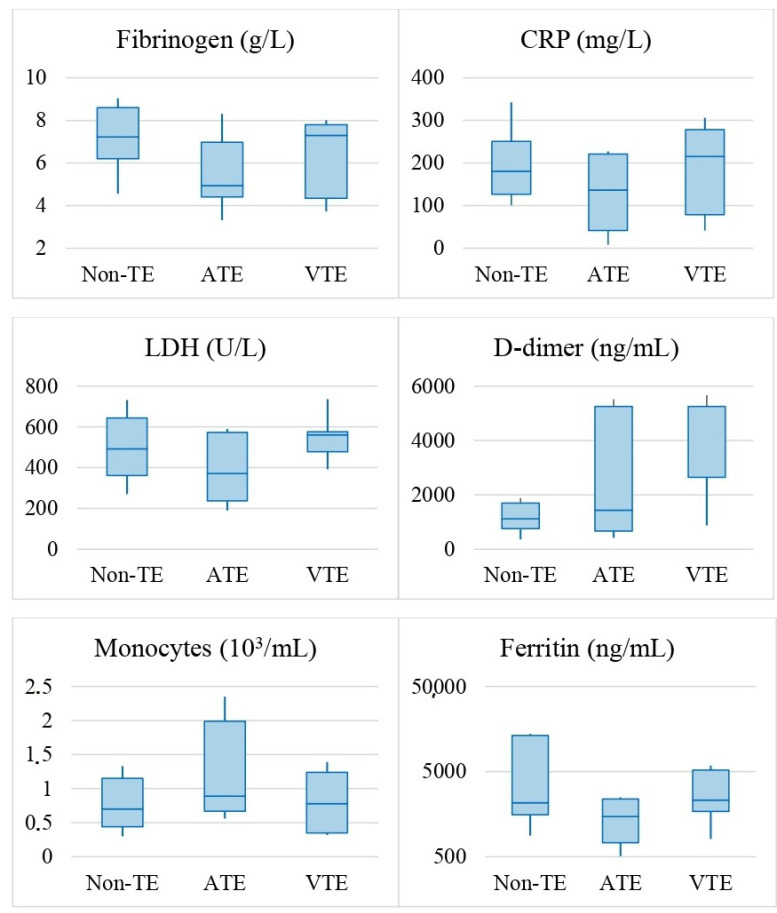
Boxplots of statistically significant laboratory parameters upon hospital admission of patients with non-TE, ATE or VTE. Comparisons were conducted using the Kruskal–Wallis H test. A *p* value of <0.05 was considered statistically significant.

These comparisons are represented in Figure 1.

Other potential mechanisms may be in play as well. Platelets have been shown to interact with monocytes in COVID-19 patients and to induce expression of tissue factor, thus promoting arterial thrombosis [18]. The higher monocyte count on ATE patients may predispose to this phenomenon and lead to a higher thrombotic risk. 

Beyond pathophysiological hypothesis, the large burden of ATE upon admission to the hospital in patients with a less prominent inflammatory profile also has clinical management implications. As these patients had no other medical reason for hospital admission related to the SARS-CoV-2 infection, one must consider ATE as part of the presentation of COVID-19. Without ATE, all these cases would have been considered either mild or asymptomatic and would be managed solely with symptom relieving therapies.

This study has some limitations. The population sample is relatively small with an inherent lack of statistical power for small effects. The disease severity stratification according to level of care is also imperfect, as the period of the study represented a moment in time of extreme pressure on health care resources. The ICU capacity was quadrupled; patients were required to receive care in the general ward that, in a normal situation, would be performed in a higher-level setting. While patient selection tried to reduce this bias, the retrospective nature of the analysis makes it impossible to secure a perfect selection. The upper limit of the D-Dimer level is capped at 5250.00 ng/mL in our laboratory, due to technical aspects of the assay. As such, the comparisons using this parameter are biased as numerical values higher than 5250.00 ng/mL are not available, which would likely accentuate the statistical differences between the selected patient groups.

## 4. Conclusions

This study offers interesting results, with an early occurrence of TE, mainly ATE, which can be the earliest presentation in COVID-19 infection, and an increased incidence of ATE over VTE. The less prominent increase in inflammatory markers in ATE patients comparatively to the other groups of COVID-19 patients with and without thrombosis, together with the early presence of the arterial event in patients with otherwise no reason for hospitalization, may also suggest, at least for ATE events, a direct role of SARS-CoV-2 in thrombotic process. This fact corroborates previous proposals that the underlying mechanism of COVID-19-associated arterial thrombosis is independent of the cytokine storm that occurs in patients with COVID-19-associated venous thrombosis [17]. Larger studies are needed to identify patients at risk of ATE upon SARS-CoV-2 infection and appropriate thromboprophylaxis to prevent these events.

## Figures and Tables

**Table 1:** Characteristics of patients with TE and the selected control group. TE: Thrombotic event; ICU: Intensive care unit; IT: Insulin Treated; COPD: Chronic obstructive pulmonary disease; HIV: Human immunodeficiency virus; HCV: Hepatitis C virus.

	TE	Non-TE
Number of patients	25	24
Male/:Female ratio	18/:6	18/:7
Age (years)	70.64 ± 13.43	72.67 ± 4.66
Patients in ICU	17	17
Co-morbidities		
Obesity	9	9
IT Diabetes Mellitus 2	1	1
Non-IT Diabetes Mellitus 2	7	6
Hypertension	14	14
Statin Therapy	9	9
Obstructive Sleep Apnoea	2	2
Asthma	1	0
COPD	1	1
Cardiac Pacemaker	1	1
Atrial Fibrillation	3	2
Atherosclerosis	4	4
Active Neoplasia	1	0
Renal Transplant	1	0
Systemic Lupus erythematosus	1	0
HIV/HCV co-infection	1	1

**Table 2:** Description of thromboembolic events.

COVID-19 Patients	N = 290
Thrombotic Events	N (%)
TE	25 (8.62%)
Isolated VTE	7 (2.41%)
Isolated ATE	16 (5.52%)
Multiple Mixed Events	2 (0.69%)
VTE	7 (28%)
Pulmonary Embolism	4
Venous Sinus Thrombosis	1
Superior Vena Cava Thrombosis	1
Deep Leg Vein Thrombosis	1
ATE	16 (64%)
Ischemic Stroke	11
ST—elevation Myocardial Infarction	5
Multiple Mixed Events	2 (8%)
TE upon hospital admission	17 (68%)
ATE	13 (52%)
VTE	4 (16%)

**Table 3:** Laboratory parameters (median values and interquartile range) upon hospital admission and comparison between groups of patients. Units and reference range are presented next to each parameter, on the left column. Comparisons were conducted using the Kruskal–Wallis H test. A *p* value of <0.05 was considered statistically significant.

Admission Levels	Non-TE	TE	ATE	VTE	TE/Non-TE	ATE/Non-TE	VTE/Non-TE	ATE/VTE
CRP (mg/L) (0–5.0)	126.44 (100.59–181.04)	77.00 (13.51–169.46)	41.61 (7.76–136.73)	78.36 (41.20–214.96)	*p* = 0.021	*p* = 0.007	*p* = 0.219	*p* = 0.204
D-dimer (ng/mL) (<500)	753.50 (362.75–1112.25)	1128.00 (451.00–5250.00)	666.00 (420.00–1428.00)	2645.00 (871.00–5250.00)	*p* = 0.049	*p* = 0.526	*p* = 0.014	*p* = 0.138
LDH (U/L) (135–214)	361.00 (270.00–491.50)	356.50 (204.25–553.75)	237.00 (189.00–371.00)	478.00 (392.00–561.00)	*p* = 0.690	*p* = 0.037	*p* = 0.130	*p* = 0.018
Ferritin (ng/mL) (2.20–178)	1555.00 (887.00–2158.00)	1219.50 (324.75–1977.50)	730.00 (299.75–1483.00)	1707.00 (808.00–2318.00)	*p* = 0.235	*p* = 0.045	*p* = 0.294	*p* = 0.205
Fibrinogen (g/L) (1.7–2.5)	6.20 (4.56–7.22)	4.63 (3.51–5.00)	4.41 (3.32–4.94)	4.35 (3.73–7.30)	*p* = 0.014	*p* = 0.010	*p* = 0.232	*p* = 0.409
Monocytes (10^3^/μL) (0.20–0.80)	0.44 (0.30–0.70)	0.64 (0.36–0.88)	0.67 (0.52–0.89)	0.35 (0.32–0.78)	*p* = 0.095	*p* = 0.033	*p* = 0.850	*p* = 0.141
Hemoglobin (g/dL) (Male:13–17) (Female:12–15)	13.95 (12.68–14.56)	13.80 (12.95–14.35)	13.50 (12.73–14.18)	14.20 (13.3–14.5)	*p* = 0.802	*p* = 0.214	*p* = 0.586	*p* = 0.076
Leucocytes (10^3^/μL) (4.00–11.00)	6.47 (5.60–8.88)	8.18 (5.05–11.25)	8.21 (6.16–10.20)	6.59 (4.86–14.08)	*p* = 0.390	*p* = 0.639	*p* = 0.850	*p* = 0.841
Neutrophils (10^3^/μL) (2.0–7.5)	5.17 (3.93–6.77)	5.98 (3.99–9.94)	6.12 (3.85–8.31)	5.88 (4.11–11.17)	*p* = 0.624	*p* = 0.858	*p* = 0.671	*p* = 0.462
Lymphocytes (10^3^/μL) (1.50–4.00)	0.93 (0.53–1.13)	0.84 (0.54–1.42)	0.87 (0.64–1.42)	0.55 (0.42–1.44)	*p* = 0.749	*p* = 0.269	*p* = 0.539	*p* = 0.256
Platelets (10^3^/μL) (150–400)	188 (157–251)	207 (163–309)	203 (163.50–301.50)	221 (133–332)	*p* = 0.503	*p* = 0.772	*p* = 0.603	*p* = 1.000
Myoglobin (μg/L) (28–72)	62.20 (40.60–218.00)	65.90 (42.85–132.50)	75.45 (40.03–200.00)	63 (45.00–82.20)	*p* = 0.735	*p* = 0.774	*p* = 0.623	*p* = 0.663
Creatine Kinase (IU/L) (24–204)	90.95 (48.25–214.55)	163.50 (107.75–294.10)	166.55 (96.78–271.23)	120.30 (105.90–190.30)	*p* = 0.158	*p* = 0.327	*p* = 0.293	*p* = 0.551

## Data Availability

The data presented in this study is not available as clinical records cannot be publicly released. For furthers queries, contact one of the corresponding authors.
